# Study of the Bioremediation of Atrazine under Variable Carbon and Nitrogen Sources by Mixed Bacterial Consortium Isolated from Corn Field Soil in Fars Province of Iran

**DOI:** 10.1155/2013/973165

**Published:** 2013-03-05

**Authors:** Mansooreh Dehghani, Simin Nasseri, Hassan Hashemi

**Affiliations:** ^1^Environmental Health Engineering Department, School of Health, Health Faculty, Shiraz University of Medical Sciences, Shiraz, Iran; ^2^Environmental Health Engineering Department, Health Faculty, Tehran University of Medical Sciences, Tehran, Iran; ^3^Environment Research Center, Isfahan University of Medical Sciences, Isfahan, Iran

## Abstract

Atrazine herbicide that is widely used in corn production is frequently detected in water resources. The main objectives of this research were focused on assessing the effects of carbon and nitrogen sources on atrazine biodegradation by mixed bacterial consortium and by evaluating the feasibility of using mixed bacterial consortium in soil culture. Shiraz corn field soil with a long history of atrazine application has been explored for their potential of atrazine biodegradation. The influence of different carbon compounds and the effect of nitrogen sources and a different pH (5.5–8.5) on atrazine removal efficiency by mixed bacterial consortium in liquid culture were investigated. Sodium citrate and sucrose had the highest atrazine biodegradation rate (87.22%) among different carbon sources. Atrazine biodegradation rate decreased more quickly by the addition of urea (26.76%) compared to ammonium nitrate. Based on the data obtained in this study, pH of 7.0 is optimum for atrazine biodegradation. After 30 days of incubation, the percent of atrazine reduction rates were significantly enhanced in the inoculated soils (60.5%) as compared to uninoculated control soils (12%) at the soil moisture content of 25%. In conclusion, bioaugmentation of soil with mixed bacterial consortium may enhance the rate of atrazine degradation in a highly polluted soil.

## 1. Introduction

Atrazine, 6-chloro-N2-ethyl-N4-isopropyl-1, 3, 5 atrazine, 4-diamine, is a selective herbicide that has been extensively used in corn production to control many broad-leaf and some grassy weeds. Atrazine has long-term reproductive and endocrine-disrupting effects and a probable human carcinogen [1]. International Agency for Research on Cancer (IARC) has concluded atrazine as a group 2B carcinogen. The maximum contaminant level (MCL) for atrazine in drinking water established by the USEPA is 3.0 *μ*gL^−1^ [2]. 

Atrazine is moderately persistent in the environment and despite its low solubility, water resources contamination, it has become an international issue [3]. The major dissipation route for atrazine is biodegradation, runoff, and leaching [4, 5]. The rate of biodegradation of atrazine is reduced due to the adsorption, and desorption, and its bioavailability is the rate-limiting step in biodegradation [6].

Bioremediation is currently used to clean a wide variety of chemicals. There are basically two approaches to bioremediation: biostimulation and bioaugmentation [7]. Bioaugmentation is the addition of acclimated indigenous bacteria that can degrade the contaminant at accelerated rates. The bacteria are isolated from the heavily polluted soil in the laboratory through the enrichment process. The enrichment culture technique developed a mixed consortium of bacteria that are able to degrade the herbicide [8]. A research was done by Dehghani and colleague confirmed that atrazine biodegradation was enhanced in Kavar corn field soil compared to the other soils that had not been exposed to the herbicide [8]. Therefore, several applications of atrazine on soil resulted in an enhancement of atrazine degradation. Biostimulation involves addition of nutrients that are required for biodegradation of a pollutant. The addition of nutrient causes an increase of microbial populations, thereby, increasing the number of indigenous microorganisms capable of degrading the pollutant [7].

N-containing compounds such as atrazine have been shown to serve as sources of nitrogen. In theory, addition of high C/N should induce nitrogen limitation and increase selective pressure for utilization of recalcitrant N sources like s-triazines, which contain N that can be used by certain bacteria and microbial consortia [9]. Cometabolic biotransformation can be enhanced by an increase in microbial activity which is stimulated by addition of organic matter [10]. In the past few decades, intensive use of agricultural fertilization and herbicides has contributed to increasing concentration of N and herbicides. 

Mixed microbial consortia [8, 11] and strains have been isolated from soils and with a capability to complete the mineralization of the ring. The *Agrobacterium* strain J_14_, *Rhodococcus erythropolis, Pseudaminobacter,* and *Nocardioides *can use atrazine as sole carbon and nitrogen source [12–14]. Laboratory experiments indicated that addition of carbon to soil inhibited atrazine biodegradation, but inorganic phosphate stimulates atrazine biodegradation [15]. Atrazine degradation rate was increased by addition of carbon sources to pure and mixed bacterial culture [16]. Ostrofsky and his coworkers (2001) found that cyanuric acid amendment increased atrazine mineralization by stimulation of general microbial population and activity [17]. In liquid culture and in the presence of simple carbon sources, 96% of the applied atrazine mineralized within 7 days. Addition of glucose enhanced atrazine mineralization due to cometabolism rather than direct metabolism [18]. However, another study showed that high nitrate concentration inhibited atrazine mineralization [19]. Topp et al. (1996) showed that the effect of N addition varies with the form and amount of added N [10]. A high concentration of inorganic nitrogen greatly decreased atrazine mineralization in soil. Atrazine mineralization in compost-amended agricultural soils was inhibited when fertilized as CaNO_3_ [20]. However, another study showed that the utilization of atrazine by *Pseudomonas* ADP in the presence of exogenous nitrogen (NH_4_, NO_3_, urea and glycine) was not affected in a great amount by the presence of exogenous nitrogen [21]. Degradation of atrazine (about 87%) by *P. ADP *and A.* radiobacter* was unaffected by the presence of N source, whereas no degradation occurred with bacterium M91-3 in media containing urea or NH_4_-N [21]. Another study reported that mineralization of atrazine by indigenous soil bacteria was inhibited by the addition of inorganic nitrogen [22]. A study showed that addition of other organic amendments containing nitrogen suppressed atrazine [23].

However, there are some limitations to the use of acclimated microbial cultures to degrade organic compound in a real field. Strains isolated in laboratory conditions might be stressed when reintroduced into the soil. Physiochemical conditions of soils, and competition with native microorganisms, may destroy or reduce the inoculums and limit its degradative capacity [24]. Brandon and his coworkers (1997) showed that atrazine biodegradation was higher in liquid cultures than soil [25]. He found that bacterial consortium in soil culture degraded 78% and 21% of atrazine at initial concentrations of 0.046 and 0.23 moles in 100 days. However, in liquid cultures, 90% and 56% of atrazine degraded in 80 days, respectively. In liquid culture, atrazine and its two dealkylated metabolites were equally degraded by the microbial consortium. The production of hydroxyatrazine was observed during atrazine catabolism [26]. 

Since Fars is an agricultural province and enjoys the top rank in wheat and corn production in the country in recent years, atrazine has been widely used as a selective herbicide to control broad-leaf and grassy weeds in agricultural corn fields. The high incidence of atrazine to contaminate water resources and the increasing concern about the toxicological properties of atrazine has made researches directed toward bioremediation of atrazine in polluted sites. Therefore, the main objectives of this research are evaluating the effects of carbon and nitrogen sources on atrazine biodegradation using mixed bacterial consortium isolated from corn field soil located in South of Shiraz (Kavar) and determining the efficiency of atrazine biodegradation process in soil culture. Kavar corn field soil with a long history of atrazine application in Fars province of Iran has been explored for their potential of atrazine biodegradation.

## 2. Materials and Methods

### 2.1. Sampling Site and Preparation

Soil samples for liquid cultures were taken from South of Shiraz (Kavar) corn field with a long history of atrazine application (more than 10 years) in Fars province. Soil samples for soil cultures were also taken from a field in Bajgah which has been under alfalfa cultivation for 3 years and has not received atrazine in the past 10 years. Disturbed soil samples were collected from 0 to 20 cm of soil depth with a hand-driven soil auger and stored at 4°C until they were used. The soil samples were air-dried and passed through 2 mm sieve to be prepared for further microbiological examinations.

### 2.2. Soil Analysis

The general physiochemical characteristics of soil were determined. Hydrometer was used to determine soil textures using the Guelph method. Other soil characteristics such as soil solution pH [27] and organic matter content [28] were determined. The soil texture in Kavar corn field was loam and the amount of sand, silt, and clay distribution were in order of 47.44%, 31.5% and 17.06%. Soil pH was 7.94. Organic matter content in soil was 8.8 g kg^−1^ soil. The native soil characteristics at Kavar site was fine loamy, mixed, thermic Typic Haploxerepts. At Bajgah field, the soil texture was clay-loam and the amount of sand, silt and clay distribution were in order of 28.7%, 33.3% and 32.0%. Soil pH was 7.5. Organic matter content in soil was 17 g kg^−1^ soil. The native soil characteristics at Bajgah site was fine loamy, mixed, and thermic, Typic Calcixerepts.

### 2.3. Chemicals and Analytical Method

All chemicals used were of reagent grade and purchased from Merck (Germany). Atrazine standard was supplied by Accua Standard Europe, Switzerland. The soil samples were transported to the laboratory in zipped plastic bags and were kept frozen at −20°C until they were ready for chemical analysis. The soil samples were thawed and air-dried at dark in room temperature and screened through a 2.0 mm sieve for maintaining homogeneity of soil in order to reduce the variability of adsorption data [29]. 30 mL of dichloromethane was added to 10 g of the soil sample and shaken in a reciprocal shaker for 20 minutes. After filtration, the organic phase was transferred to a separating funnel and then atrazine was back extracted with 20 mL HCl (0.01 N). Afterwards, the liquid phase was collected and transferred to a 15 mL glass vial and stored in a refrigerator prior to electrochemistry analysis. Square wave voltammetry with the Hanging Mercury Drop Electrode (Auto Lab type analyzer equipped with Metrohm VA STAND 663 and GPES 4.9 software) was used in this study to determine atrazine residual concentration in soil samples [30]. Atrazine recovery percent from soil with this method of extraction was 98%.

### 2.4. Enrichment Culture

In order to isolate mixed bacterial consortiums capable of growth on atrazine as a carbon source, the selective enrichment culture and basal salt medium were prepared as described in Rousseaus, [31]. Ten grams of wet soil was inoculated into 90 mL of atrazine medium and supplemented with sodium citrate and Delvocid (25 mg L^−1^) after autoclaving [31]. Delvocid was used to prevent the growth of fungi and pH was also adjusted to 7.5. Cultures were incubated aerobically on a reciprocal shaker (150 rpm) at room temperature in dark to preclude photolysis reactions. All enrichment cultures were subcultured on the same medium at one-week interval. From a one-week-old culture, 10 mL was transferred to 90 mL of freshly prepared atrazine medium. After culture was subculture for 30 and 300 days under conditions of nitrogen limitation, the remained atrazine after inoculation of the media for 10 days was quantified by electrochemistry. Controls contained 1 g of sodium azide per liter as growth inhibitor. The bacterial consortium was harvested by centrifugation (6000 g at 40°C for 20 minutes) washed twice with 0.1 mL phosphate buffer (pH = 7.3). 

### 2.5. Effects of Carbon and Nitrogen Sources on Atrazine Biodegradation

Different carbon compounds such as glucose (G), sodium citrate (SC), sucrose (SU), starch (ST) with three replications each at a concentration of 2 g L^−1^, and also the combination of these mentioned carbon sources such as G + SC, ST + SU, SU + SC, ST + SC, SU + G, and ST + G each at a total concentration of 2 g L^−1^ were added to atrazine mineral salt broth. After inoculation of the media supported with carbon sources, they were incubated at room temperature and placed in dark for 10 days. Control and blank without bacteria inoculation and no carbon sources (NCS) were also used for this study. After 10 days, the remained atrazine was measured.

To measure the influence of nitrogen sources on the efficiency of atrazine biodegradation by the bacterial consortium in liquid culture, nitrogen sources as routine fertilizers were added to atrazine mineral salt broth containing sucrose and sodium citrate. Ammonium nitrate and urea as fertilizers were applied to corn field at a concentration of 600–825 and 200–400 kg ha^−1^, the nitrogen percent for these fertilizers were 34% and 46%, respectively. Urea was added to atrazine minimal salt media at a concentration of 138–690 mg L^−1^ as N which was corresponded with 100–500 kg ha^−1^. Ammonium nitrate was added to atrazine media at a concentration of 170–306 mg L^−1^ as N which was corresponded with 500–900 kg ha^−1^. After inoculation of the media supported with nitrogen sources, they were incubated at room temperature in dark for 10 days. Three replications were done for each nitrogen source. Control and blanks without bacterial inoculation and nitrogen source were used for this study. After 10 days, the remained atrazine reduction without inoculation was less than 3% [9].

A laboratory experiment was arranged to measure the influence of pH on the efficiency of atrazine biodegradation by the bacterial consortium in liquid culture. A different pH from 5.5 to 8.5 with three replications was used in atrazine mineral salt broth containing sucrose and sodium citrate and no nitrogen sources. Control without bacterial inoculation was used for this study.

### 2.6. Effect of Initial Atrazine Concentration and Soil Moisture on Atrazine Biodegradation in Soil

Atrazine degradation rate by the bacterial consortium was measured in 100 mL capped Erlenmeyer flask containing soil samples. Ten grams of soil sample was brought to the desired soil moisture (7% and 25%) by the addition of sterile deionized water. The experimental design consisted of 36 flasks with 12 treatment and three replications for each treatment. Normal application rates for atrazine are 0.5–2.5 kg ha^−1^ (0.3–1.5 kg a. i. ha^−1^). The initial atrazine concentrations of 1.3 and 6.7 mg g^−1^ soil are corresponded to 0.5 and 2.5 kg ha^−1^, respectively. However, atrazine concentration of 20 mg g^−1^ soil is related to 7.5 kg ha^−1^(4.43 kg a. i. ha^−1^) which is considered a relatively high atrazine concentration and might have occurred due to an accidental spillage. After one day of incubation to allow herbicide sorption to the soil, inoculation with 300 *μ*L phosphate buffer containing the selected bacterial consortium was added to soil to yield 7.5 × 10^5^ bacterial cell g^−1^ soils as determined by plating on soil extract agar. A noninoculated control received only a sterile phosphate buffer. The soil samples were mixed until they were homogeneously wet and then incubated at room temperature in dark until the end of the experiment. Soil samples were extracted after 30 days of incubation and analyzed with electrochemistry. Soil moisture was maintained constant through the incubation by weighing and correcting for any weight loss by adding sterile deionized water. To maintaining high population of atrazine degraders, the inoculation with the consortium was made every 5 days over a 30-day period for a total of 6 inoculations [32]. Soil samples at the initial atrazine concentration of 6.7 mg g^−1^ soil were extracted at the incubation time of 2, 10, 20, and 30-day and analyzed to determine the remained atrazine concentration at each time interval. 

## 3. Results

### 3.1. Atrazine Biodegradation Rate and the Effects of Different Parameters

**Figure 1:**
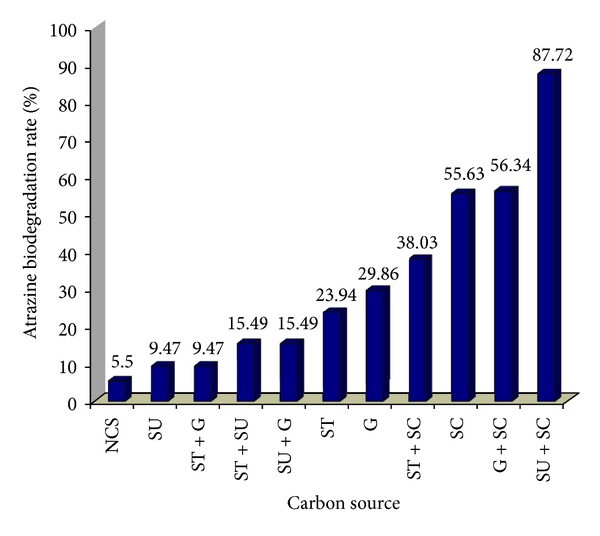
Atrazine biodegradation rate Kavar corn field bacterial consortium at different carbon sources.

By using a mixed bacterial consortium with a high capability of atrazine degradation isolated from Kavar corn field soil, the effect of different carbon sources on atrazine biodegradation was studied (Figure 1). According to this Figure, the percentage of atrazine biodegradation rate for different carbon sources was in the range of 9.47% to 87.72%. Blank and control without carbon source and bacteria inoculation were also used. A blank sample did not adequately support the growth of consortium bacteria (due to low turbidity), and the rate of atrazine degradation is lass than 3%. Linear regression showed that there was a significant difference between different carbon sources and atrazine biodegradation rate (*P* < 0.001). 

**Figure 2:**
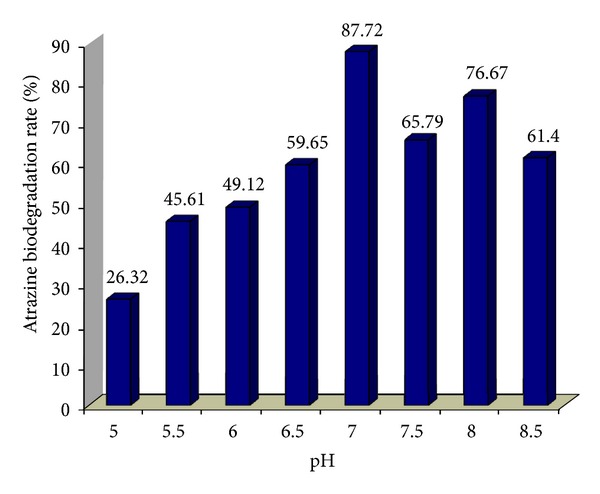
Atrazine biodegradation rate by Kavar corn field bacterial consortium at a different pH.

The effect of different nitrogen sources on atrazine biodegradation was shown in Tables 1 and 2. According to Table 1, the percent of atrazine biodegradation rate decreased from 87.72% to 29.58% as the ammonium nitrate concentration increased from 0.0 to 900 kg ha^−1^ soil. The rate of atrazine biodegradation for ammonium nitrate decreased quickly when the concentration of ammonium nitrate increased from zero to 600 kg ha^−1^ soil, after that the reduction rate of atrazine degradation was getting slower. The same trend has been observed for atrazine biodegradation in the presence of urea. According to Table 2, the percentage of atrazine biodegradation rate decreased from 87.72% to 26.76% as the urea concentration increased from 0.0 to 500 kg ha^−1^ soil. For urea, an initial slope was 0.21 and then the slope became 0.01. Atrazine biodegradation rate under nitrogen amendment showed an initial sharp decreasing slope and then reaching constant with relative slower rate. Variations of pH on atrazine degradation rate were examined when sucrose and sodium citrate were used as a carbon source, while there was no nitrogen source available (Figure 2). According to regression analysis, it can be concluded that there was a significant difference between pH and atrazine biodegradation (*P* < 0.05). 

### 3.2. Effect of Initial Atrazine Concentration and Soil Moisture on Atrazine Biodegradation in Soil

**Figure 3:**
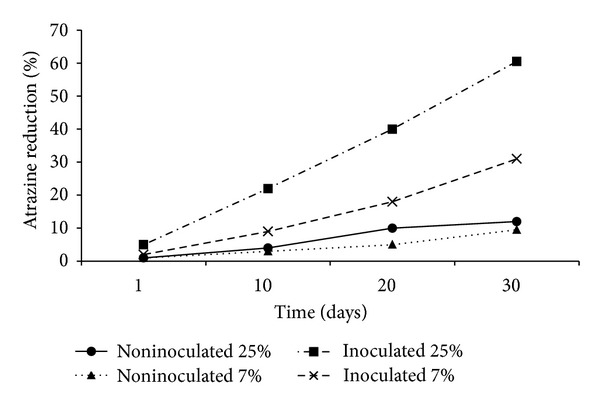
The percentage of atrazine reduction for noninoculated and inoculated soil at two different soil relative moisture contents (SRMC).

Table 3 showed the effect of initial atrazine concentration and soil moisture on the biodegradation of the inoculated soil. According to data on this table, after 30 days of incubations atrazine degradation rate for noninoculated soil samples was low and atrazine reductions were only 7% to 19%. However, for inoculated soil samples, degradation rate was higher and its reductions were 19.5–72%. The inoculated soil samples at 25% soil moisture at initial concentration of 1.3, 6.7, and 20 mg g^−1^ soil, atrazine reductions were 69.5%, 60.5% and 30.5% within 30 days of incubation, respectively.  Figure 3 showed that atrazine biodegradation rate during the different time intervals in soil culture.

**Figure 4:**
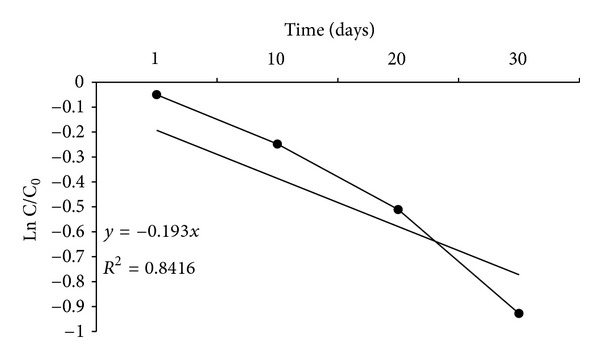
Semilogarithmic plot of concentration/initial concentration (C/C_0_) of biodegradation of atrazine by mixed bacterial consortium over time.

Figure 4 depicted the plot of the semilogarithm of initialized atrazine concentration (C/C_0_) versus time. During the 30 days of incubation period, atrazine concentration decreased from an initial concentration of 6.7 mg g^−1^soil to a final concentration of 2.67 mg g^−1^soil. Only a 5% decrease in atrazine concentration was seen by the first day of the incubation period. However, Figure 3 showed that at 20 days 40% of the atrazine had degraded. After that a very slow increase in atrazine degradation was observed and remained constant until the end of the incubation period. Assuming a pseudo-first-order reaction for the disappearance of atrazine, a plot of the natural logarithm of initialized atrazine concentration (C/C_0_) versus time resulted in a rate constant equal to 0.0328 d^−1^ (Figure 4). The *t*_1/2_ for atrazine calculated from the plot was 21.13 d. The initial slow degradation rate of atrazine was followed by a much faster degradation rate that lasted about 5 day and then the degradation rate became slower and finally remained constant until the end of incubation period.

## 4. Discussion

Carbon sources of sodium citrate and sucrose had the highest atrazine biodegradation rate. According to data, it is clear that sodium citrate had the major role in atrazine degradation. However, sucrose as the only carbon source had resulted lowest atrazine degradation. Mandelbaum and his coworkers (1993a) used sodium citrate and sucrose as the carbon source [9]. According to data in this research, the percentage of atrazine biodegradation rate with no carbon sources was only 5.5%. The presence of additional substrates could initiate cometabolism of atrazine. Past studies showed that carbon in atrazine ring cannot be used by many bacteria as energy sources; however, carbon in alkylated group provided carbon for bacterial growth. The low amount of atrazine cannot support bacterial growth, and therefore, a supplemental carbon was needed [33]. 

According to data on Tables 1 and 2, cells grown on exogenous nitrogen source have failed to reduce supernatant atrazine concentration significantly. Atrazine utilization is repressed under nitrogen-sufficient growth condition and activated under nitrogen limitation. Therefore, atrazine catabolism is repressed when another nitrogen source is available. Many investigations also showed a negative effect of nitrogen amendment on atrazine biodegradation by indigenous populations in soils [34, 35]. Many researchers noted a high concentration of mineral nitrogen greatly decreased mineralization in soil [36]. However, other study reported that organic nitrogen supplied in dairy manure increased atrazine mineralization [10]. Data regarding the effect of pH shows that as pH increased from 5.0 to 7.0, the rate of atrazine biodegradation increased. However, as pH increased from 7.0 to 8.5 this caused a reduction rate in atrazine biodegradation. Showing pH of 7 is optimum for atrazine biodegradation. The biodegradation rate was 87.72% in this case.

Atrazine biodegradation rates were significantly enhanced in the inoculated soils as compared to uninoculated control soils. After 30 days, the percent of atrazine reduction was only 12% for uninoculated soils at initial atrazine concentration of 6.7 mg g^−1^ soil and the soil moisture of 25%. However, soil that was inoculated every 5 days with the bacterial consortium had reduced atrazine up to 60.5% at the same initial atrazine concentration and the soil moisture by day 30 (Table 3). As initial atrazine concentration increased from 1.30 to 20 mg g^−1^ soil, the percentage of atrazine reduction decreased from 69.5% to 30.5% (inoculated soils and soil moisture 25%). The decrease in atrazine reduction at 20 *μ*g g^−1^ soil was possibly due to the result of complex interaction between microbial activity and nutrient availability. Therefore, unbalanced nutrient supply and not atrazine toxicity was probably responsible for the decrease.  Figure 3 showed that atrazine degradation rate increased as time passed. According to (Table 3), enhanced atrazine biodegradation rates occurred with an increase in soil moisture from 7% to 25%. The percent of atrazine reduction increased from 32.5% to 69.5% as the soil moisture increased from 7% to 25% (at initial atrazine concentration of 1.30 mg g^−1^ soil). Many researchers found that higher atrazine biodegradation rate occurred with an increase in soil moisture. Soil moisture influences microbial processes through direct effect (e.g., water availability) or indirect effects, for example, solute diffusion, chemical availability, and aeration [37]. Therefore, the positive effect of increasing soil moisture was probably due to increased microbial mobility, solute diffusion, and chemical availability which all had an indirect effect on atrazine degradation. In this study, more than 60% of atrazine was degraded in 30 days of incubation periods (Figure 3). Atrazine degradation exhibited a half-life of approximately 21.13 d.

In conclusion, our results confirmed that atrazine biodegradation was higher in liquid cultures than soil. The mixed bacterial consortium in soil culture degraded 69.5% of atrazine at initial concentrations of 1.3 mg g^−1^ soil in 30 days. However, in liquid cultures, 87.72% of all atrazine degraded in 10 days. Results of this study suggested that atrazine bioremediation in soil utilizing atrazine-degrading bacterial consortium could be accomplished across a wide range of atrazine concentration if the soil moistures swere enough and also nutrient availability was balanced, too. The bacterial consortium with the ability to degrade atrazine provided a good opportunity for increasing the degradation rate of this herbicide through bioaugmentation. It is very important to note that these bacteria in the controlled laboratory conditions (with the addition of carbon and nitrogen sources and without the interaction of environmental factors) had been able to use atrazine. However, there are some limitations to the use of acclimated microbial cultures to degrade the herbicide in a real field. Therefore, it is highly recommended that the feasibility of atrazine degradation to be studied by the bioaugmentation of mixed bacterial isolates to the real contaminated soil. The mixed bacterial consortium successful in laboratory studies may fail in the real field because of their sensitivity to high concentrations of other compounds. 

## Figures and Tables

**Table 1:** Atrazine degradation rate, (%) by Kavar corn field bacterial consortium at different ammonium nitrate concentrations (kg ha^−1^).

Ammonium nitrate (kg ha^−1^)	Atrazine degradation rate, (%)			Mean	SD
	Replication 1	Replication 2	Replication 3
0	88.16	88.00	87.00	87.72	0.63
500	74.95	76.00	73.00	74.65	1.52
600	46.82	44.00	41.00	43.94	2.91
800	35.64	36.90	39.00	37.18	1.70
900	27.74	31.00	30.00	29.58	1.64

**Table 2:** Atrazine degradation rate (%) by Kavar corn field bacterial consortium at different urea concentrations (kg ha^−1^).

Urea (kg ha^−1^)	Atrazine degradation rate (%)			Mean	SD
	Replication 1	Replication 1	Replication 1
0	90.16	85.00	88.00	87.72	2.06
100	73.00	65.00	60.00	68.00	4.36
200	47.30	41.00	42.80	43.70	3.25
300	23.40	26.90	25.00	25.10	1.75
500	28.20	26.02	26.15	26.76	1.22

**Table 3:** The effect of inoculation of the bacterial consortium, initial atrazine concentration (*μ*g g^−1 ^soil), and soil moisture (%) on atrazine reduction (%) after 30 days of incubation time in soil.

Replication	Atrazine reduction, %											
	Inoculation (+)						Inoculated (−)					
	Initial atrazine concentration, (*μ*g g^−1^ soil)											
	1.30		6.70		20.0		1.30		6.70		20.0	
	Soil moisture, (%)											
	7	25	7	25	7	25	7	25	7	25	7	25
1	33.0	72.0	30.0	61.0	19.5	31.0	14.0	16.0	10.0	12.0	7.0	9.0
2	35.5	67.5	27.0	58.0	21.0	32.0	10.5	19.0	10.5	14.0	8.5	9.0
3	29.0	69.0	36.0	62.5	19.5	28.5	13.0	16.0	8.0	10.0	7.0	12.0

Mean	32.5	69.5	31.0	60.5	20.0	30.5	12.5	17.0	9.5	12.0	7.5	10.0
SD	2.33	1.66	3.33	1.66	0.66	1.33	1.33	2.00	1.00	1.33	0.66	1.00
